# Clinical outcomes of CyberKnife stereotactic body radiotherapy for peripheral stage I non-small cell lung cancer

**DOI:** 10.1007/s12032-015-0506-1

**Published:** 2015-02-01

**Authors:** Ze-Tian Shen, Xin-Hu Wu, Bing Li, Xi-Xu Zhu

**Affiliations:** Department of Radiation Oncology, Jinling Hospital, Medical School of Nanjing University, Nanjing, People’s Republic of China

**Keywords:** NSCLC, CyberKnife, SBRT, Clinical outcomes

## Abstract

The aim of this study was to evaluate the clinical outcome of CyberKnife stereotactic body radiotherapy (SBRT) for patients with stage I non-small cell lung cancer (NSCLC). Fifty patients with peripheral stage I NSCLC who refused surgery or were medically inoperable were treated with 48–60 Gy (median dose: 57 Gy) in three divided doses. Histopathology was available in 86 % of patients. Thirty patients had a T1 tumor, and 20 patients had T2 tumors. More than 95 % of the target volume was covered by the 72 % isodose surface. Fiducials were implanted in or near the tumors in all patients to track tumor movement and breathing patterns. The median follow-up time was 35 months (3–45 months). Based on computed tomography scans, 40 patients achieved complete remission, six patients achieved partial remission, two patients exhibited stable disease, and two patients had progressive disease. The local control rate (CR + PR) was 92 %, and the 2-year disease control rate (CR + PR + SD) was 96 %. Overall survival for the whole group was 86 % at 1 year and 74 % at 2 years. Grade III toxicity occurred in two patients (4 %) after marker placement. Treatment-related late grade III toxicity occurred in five patients (10 %). Toxicities greater than grade III were not observed. CyberKnife SBRT achieves a high rate of local control and long-term curative effect with acceptable toxicity for patients with inoperable stage I NSCLC. However, long-term follow-up is necessary to evaluate survival and late toxicity.

## Introduction

Surgical resection is the primary treatment for early non-small cell lung cancer (NSCLC). However, radiotherapy is the only possible method for treating elderly patients with poor cardiopulmonary function, with poor body condition, or with surgical reluctance. Considering lung cancer is easily affected by respiratory motion, the area needs to be increased to avoid any leakage during tumor irradiation. However, because of numerous restrictions, the radiation dose for tumors is maintained at 60–70 Gy, which leads to a low control rate [1]. Thus, the 2- and 5-year survival rates of early peripheral stage I NSCLC are always maintained at about 39 and 13 % [2], respectively, which are far below the surgical efficacy [3, 4].

As a new means of radiotherapy, CyberKnife stereotactic body radiotherapy (SBRT) avoids the errors caused by respiratory movement through synchronous respiratory tracking technology. By implanting gold fiducials in or around the tumors, tumor movement synchronized with respiratory motion can be truly tracked, more accurately giving the tumor higher doses, while reducing the dose to normal tissues [5–8].

The planning target volume (PTV) and the volume of normal lung tissues irradiated by high dose are significantly reduced because of the high degree of conformity of the tumor target under CyberKnife SBRT. Thus, the incidence of radiation pneumonitis decreases significantly. The purpose of this study was to report and to analyze the preliminary clinical efficacy of 50 patients with stage I NSCLC.

## Materials and methods

### Ethics statement

The study was approved by Ethic Committee of Jinling Hospital. Patients have provided their written informed consents to receive the CyberKnife SBRT.

### Patients

A total of 50 patients with early (T1–T2a, stage I) peripheral NSCLC were treated at Radiotherapy Center, Jinling Hospital between March 2009 and September 2010. Peripheral lung cancer was defined as the distance from tumor to bronchus, and main bronchus in the CT was at least 2 cm. All patients were confirmed by two chief thoracic surgeons. All patients refused surgery or had no surgical indications. Up to 42 patients were diagnosed through histopathology, whereas eight patients were diagnosed through typical clinical manifestations and positron emission tomography/computer tomography (PET/CT) imaging. The patient details are shown in Table 1.

**Table 1:** Clinical data of the 50 NSCLC patients before CyberKnife radiosurgery treatment

Item	Cases (%)
*Gender*	
Male	28 (56)
Female	22 (44)
*Age* (*years*)	
>70	30 (60)
≤70	20 (40)
*Tumor position*	
Left upper lobe	7 (14)
Left lower lobe	9 (18)
Right upper lobe	15 (30)
Right middle lobe	6 (12)
Right lower lobe	11 (22)
Other	2 (4)
*Clinical stage*	
Stage IA	30 (60)
Stage IB	20 (40)
*Maximal diameter*	
≤2 cm	13 (26)
>2 cm but ≤3 cm	17 (34)
>3 cm but ≤5 cm	20 (40)
*Histology*	42 (84)
Adenocarcinoma	19 (45.2)
Squamous cell carcinoma	17 (40.5)
Large cell carcinoma	4 (9.5)
Undifferentiated carcinoma	2 (4.8)
No biopsy or inconclusive biopsy	8 (16)
*Karnofsky performance status*	
60	5 (10)
70	20 (40)
80	15 (30)
90	10 (20)
*FEV1* (*L*)	1.26 (0.75–3.28)
% Predicted FEV1	57 (23–86)

### Fiducial marker placement

All 50 patients were treated using a CyberKnife SBRT system (Accuray, USA). All patients were treated via respiration synchronous tracking (synchrony), and one to three markers (size of 6.0 mm × 8.0 mm) were required to be implanted within or around the tumor using a CT-guided 19-G needle. CT scan was performed to observe whether the markers were in the proper positions or to detect the presence of pneumothorax within 2 h after implantation. A CT scan was performed again at 7–10 days after implantation. At this time, local hemorrhage and edema subsided around the markers, whereas the markers were relatively stable and no longer moved.

### Position and target delineation

Patients were in the supine position with the body fixed with a vacuum pad. Spiral CT (Sensation 16 PET/CT, Siemens, Germany) scanning was conducted with a slice thickness of 1 mm. Pulmonary scans covered 15 cm above and below the lesions. The gross target volume (GTV) and PTV were determined according to the tumor volume. The GTV was contoured using lung window settings. We added a 8-mm margin to the GTV to account for microscopic tumor extension and residual inaccuracy of synchrony. The prescription dose was defined as 100 % of the GTV dose. The total PTV dose was not less than 80 % of the prescription dose.

### Treatment mode and methods

Before treatment, a respiratory monitoring device was used to detect the position of the infrared generator placed on the chest of the patient to create a dynamic respiratory rhythm. The X-ray kV digital images were obtained at different time points during the respiratory cycle to obtain the dynamics model between the gold seed fiducial (tumor) position and respiratory rhythm. Then, the respiratory model was used to guide the accelerator to track the lesions within the lung and to administer the dynamic radiation. The prescription dose of lesions was 48–60 Gy (median dose 57 Gy) in three divided doses. The lesions were treated once per day (rest on Saturdays and Sundays), and the total treatment time was 3–5 days (median 4 days). The equivalent biological dose was 104–150 Gy when the *α*/*β* value was equal to 10. About 130 beams were shot out through 2–3 collimators (size 20–60 mm). The median treatment time was 70 min (ranging from 40 to 130 min) at a dose rate of 400 MU/minute. During the treatment, antiemetic, dehydration, appetite improvement, and other appropriate symptomatic treatments were given when complications such as nausea, vomiting, fatigue, and anorexia occurred.

### Follow-up and appraisal

A chest CT scanning was performed during the 1st, 3rd, 6th, 9th, 12th, 15th, 18th, 21st, and 24th month within 2 years after treatment. The Response Evaluation Criteria in Solid Tumors, version 1.1 (RECIST1.1) was used to evaluate treatment efficacy [9]. The Radiation Therapy Oncology Group (RTOG) radiation injury-grading criterion was used to evaluate radiation injury. Follow-up was performed every 3 months for a total of 3–45 months, with a median follow-up of 35 months, and the last follow-up time was in February 2013.

Local recurrence was defined as a 20 % increase in CT tumor dimension compared with the previous CT scan. In addition, a PET scan was performed to assist in the diagnosis. The local control was calculated from the first day of treatment until local recurrence was diagnosed. Patients without a local recurrence were censored on the last day of contact. Overall survival was measured from the start of CyberKnife treatment until death of any cause. Cause-specific survival was measured from the start of CyberKnife treatment until death by lung cancer. Patients alive until the last date of contact were censored.

### Statistical process

SPSS 13.0 statistical software was applied for data analysis. The Kaplan–Meier method was used to analyze local control and overall survival. The log-rank method was used to test the significance compared with the survival curves. The survival time was from the date when CyberKnife was used in the treatment. *P* value <0.05 was considered statistically significant.

## Results

### Dosimetry index

**Fig. 1:**
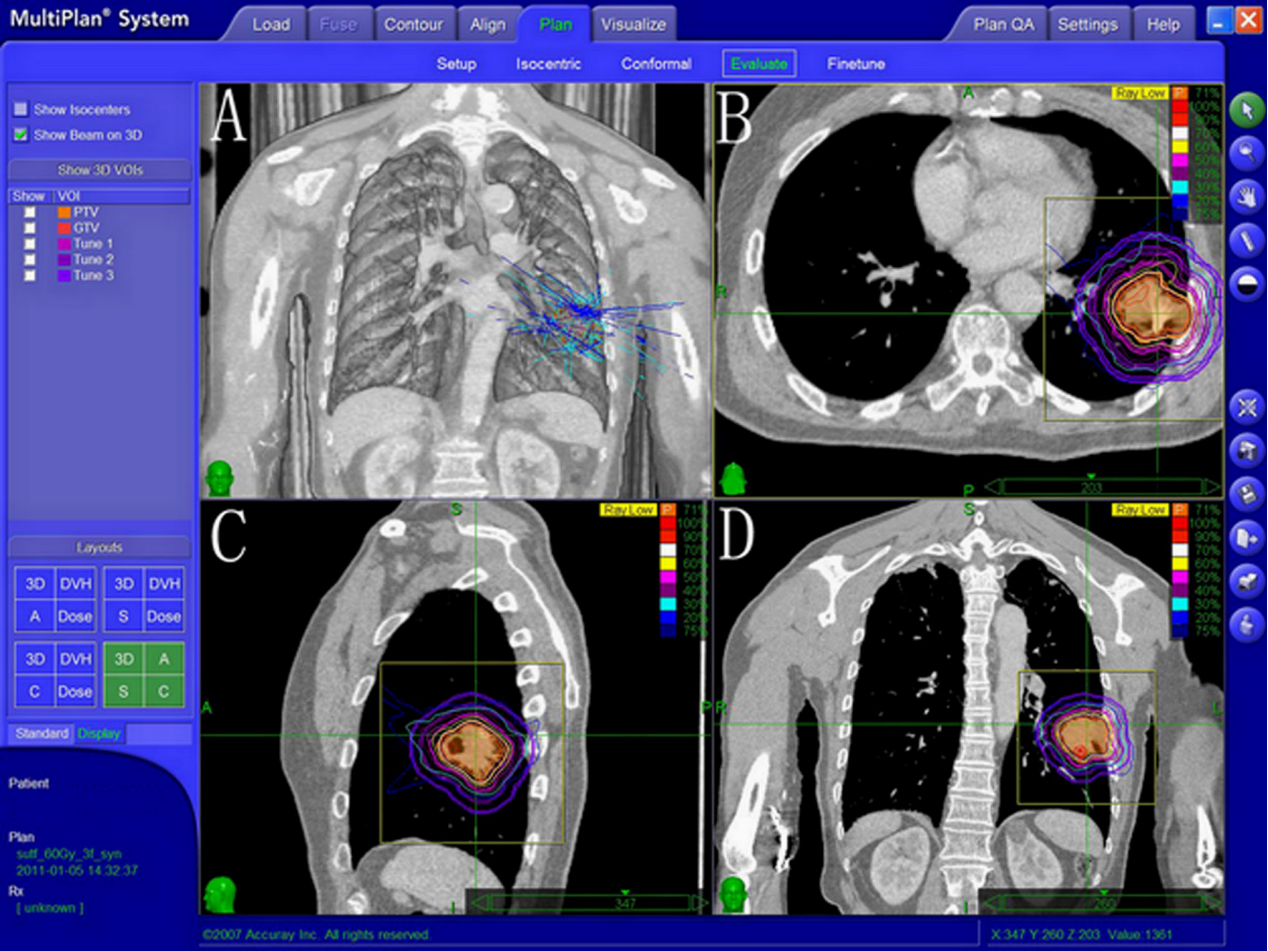
Dosimetry index of one patient with stage IB NSCLC during CyberKnife radio surgery treatment. **a** Multiple pencil beams delivered by the CyberKnife converging at the tumor target. *Isodose curves* (**b** axial view; **c** sagittal view; **d** coronal view) depict the dose distribution around the tumor volume, represented by the *red line*. The *white line* shows the 70 % *isodose line*, the *peach blossom line* is the 50 % *isodose line*, and the *light blue line* is the 20 % *isodose line*

Among 50 patients, the tumor PTV ranged from 3.4 to 166.3 cm^3^, with a median value of 27 cm^3^. The isodose level of prescription dose in the treatment plan was from 72 to 88 %, with a median of 78 %. The number of radiation fields ranged from 150 to 200 of the non-coplanar fields. The treatment plan showed that the mean Conformity Index (CI) of the pancreatic lesions in all patients was 1.17, and the mean New Conformity Index (nCI) was 1.28, which is shown in Table 2. The standard of dose limitation in critical structures is shown in Table 3. Figure 1 shows the dosimetry indicators in the treatment plan of a patient with stage IB disease (the prescription dose of 75 % isodose level was 60 Gy/3 fractions. The PTV was 58.7 cm^3^, the CI of lesion was 1.21, nCI was 1.33, and the coverage rate was 99.73 %).

**Table 2:** Dosimetry index of the 50 patients during CyberKnife radiosurgery treatment

Item	Median (range)
Tumor diameter (cm)	2.7 (1.0–5.0)
Gross tumor volume (cc)	15.6 (1.0–125)
Prescription dose (Gy)	57 (48–60)
Conformity Index (CI)	1.17 (1.07–1.30)
New Conformity Index (nCI)	1.28 (1.14–1.35)
Coverage (%)	96 (85–100)
Planned tumor volume (cc)	27 (3.4–166.3)
Prescription isodose line (%)	78 (72–88)

Conformity Index (CI): The ratio of the tissue volume that received the prescription isodose or more to the tumor volume receiving the prescription isodose or more

New Conformity Index (nCI): The data of the CI multiplied by the ratio of the total tumor volume to the tumor volume receiving the prescription isodose or more

Coverage: The volume of the tumor that received greater than or equal to the prescribed dose divided by the total volume of the tumor times 100

**Table 3:** Standard of dose limitation in critical structures

Critical structures	Dose constraints	
	Volume	Dose (Gy per fraction)
Esophagus	Any point	9
Trachea and main bronchus	Any point	10
Spinal cord	Any point	6
Plexus brachialis	Any point	8
Liver	Any point	20
Lung (right and left)	<10 %of the total volume	6.67

Dosis constraints for critical structures were taken three fractions as a standard

### Situations of treatment-related complications

#### Gold seed fiducial marker implantation before treatment

All patients were treated with respiration synchronous tracking technique and were implanted with the markers within or around the tumor through CT guided before the treatment. A total of 112 markers were implanted with the average quantity of 2.24. One marker was implanted when the tumors were ≤2 cm and with an irregular shape. If the tumors were greater than 2 cm and smaller than 3 cm, two markers were implanted. If the tumor was >3 cm and with an irregular shape, three markers were implanted. Grade III complications occurred in three cases of lung puncture. Among them, two cases developed pneumothorax caused by punctures and recovered after 5 days of closed thoracic drainage. One patient with serious hemoptysis recovered through interventional treatment and completed the treatment smoothly. Five cases developed grade II complications with <30 % pneumothorax who recovered within 2 days via suction through a fine-needle puncture. Eight cases developed grade I complications, consisting mostly of mild pneumothorax, local pain, and hemorrhage.

### Treatment of related complications

During the CyberKnife SBRT, 32 patients developed fatigue and anorexia, whereas the others had no significant symptoms. Four cases had grade II bone marrow depression, whereas 16 cases had grade I bone marrow depression. All cases returned to normal after granulocyte colony-stimulating factor (G-CSF) treatment. After the treatment, five cases developed grade III complications during follow-up, with two cases of radiation pneumonitis that needed hormonal and antibiotic treatment. The cases recovered after 3 weeks. Three cases had pain in the chest wall that required morphine for relief. Three cases developed grade II radiation pneumonitis, whereas eight cases developed grade I limited radiation pneumonitis, all of which were localized. After symptomatic treatment, the cases recovered smoothly. The side effects of all the patients are shown in Table 4.

**Table 4:** Side effects of 50 patients with NSCLC in CyberKnife radiosurgery treatment (%)

	Classification in WHO and RTOG				
	0	1	2	3	4
*Acute side effects*					
Leukopenia	35 (70)	12 (24)	3 (6)	0 (0)	0 (0)
Thrombocytopenia	45 (90)	4 (8)	1 (2)	0 (0)	0 (0)
Hemoglobin↓	47 (94)	2 (4)	1 (2)	0 (0)	0 (0)
Fatigue	18 (36)	32 (64)	0 (0)	0 (0)	0 (0)
Anorexia	30 (60)	20 (40)	0 (0)	0 (0)	0 (0)
*Late side effects*					
Pain	40 (80)	5 (10)	2 (4)	3 (6)	0 (0)
Radiation pneumonia	37 (74)	8 (16)	3 (6)	2 (4)	0 (0)

### Short-term efficacy

**Fig. 2:**
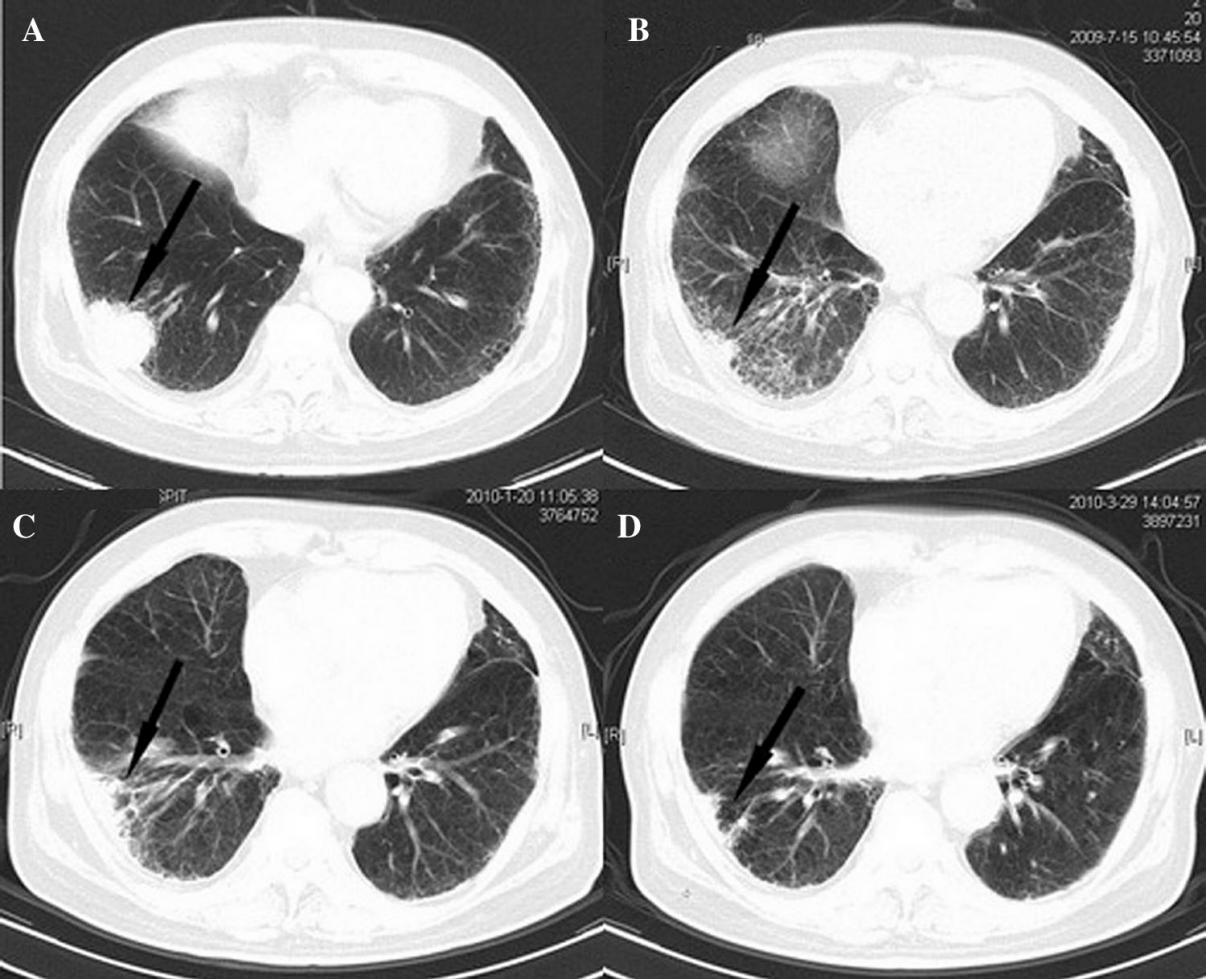
**a** Enhanced CT image of a patient with stage IB NSCLC before CyberKnife radiosurgery treatment. The arrows show the tumor before Cyberknife radiosurgery treatment; **b** The tumor volume decreased significantly at almost 1 month after CybernKnife radiosurgery tratement; **c** The tumor volume decreased much more significantly at almost 6 months after Cyberknife radiosurgery treatment, but with some inflammation; **d** The tumor was destroyed and almost disappeared at 9 months after CyberKnife radiosurgery treatment

All patients completed the treatment successfully, and all of the 50 cases were evaluated after the treatment. The results show that 40 cases had CR, six cases had PR, two cases had SD, and two cases had PD. The efficiency (CR + PR) rate of tumor was 92 %, and the disease control rate (CR + PR + SD) was 96 % in 2 years. During the follow-up period, the disease control rate for stage IA was 95 %, whereas the 2-year disease control rate was 90 %. All local recurrences were seen among patients with T2 tumors. Figure 2 shows the CT scan from 1 to 9 months after treatment in a patient with stage IB disease.

### Long-term efficacy and survival situation

**Fig. 3:**
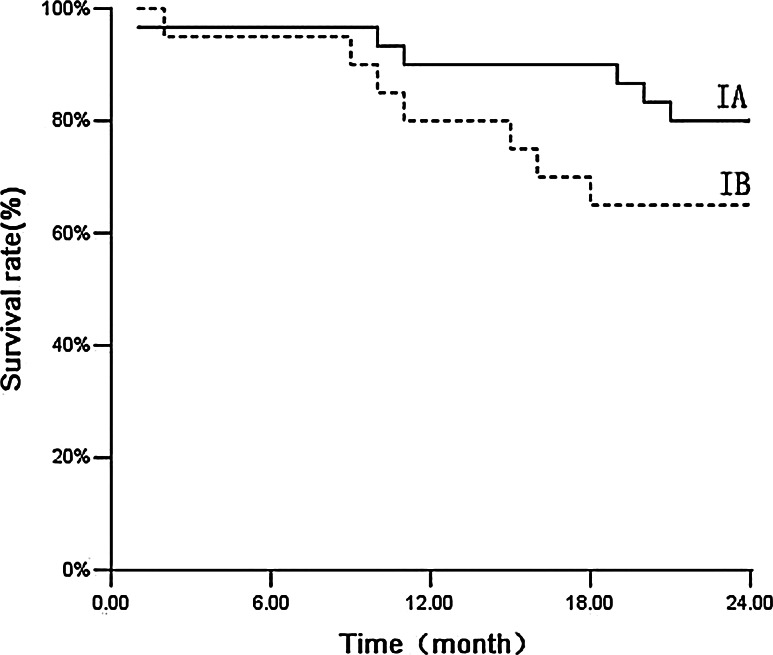
Overall survival of patients. Overall survival of patients with stage *IA* (*n* = 30) and stage *IB* tumors (*n* = 20) treated with real-time tumor tracking using CyberKnife (*P* = 0.203)

**Fig. 4:**
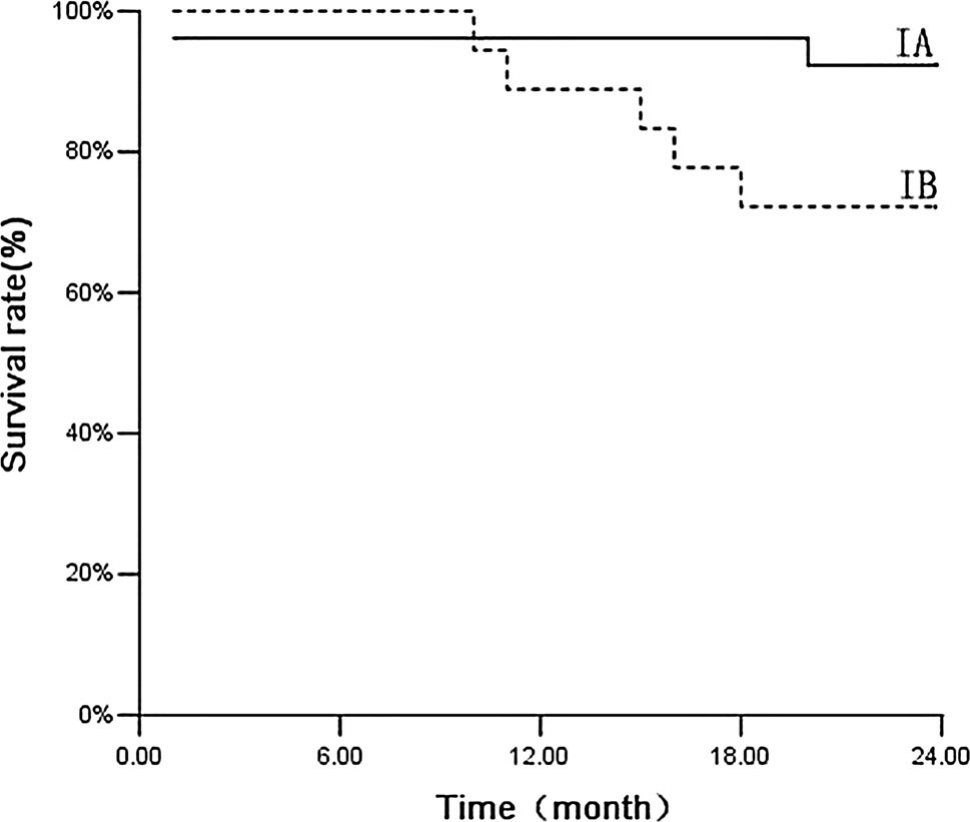
Cause-specific survival of patients. Cause-specific survival of patients with stage *IA* (*n* = 30) and stage *IB* tumors (*n* = 20) treated with real-time tumor tracking using CyberKnife (*P* = 0.07)

Among the 50 patients, the median follow-up time was 35 months and the follow-up rate was 100 %. Three cases developed local recurrence, six cases developed distant metastasis, and four cases developed both local recurrence and distant metastasis. Up to 16 patients died during follow-up: Four patients died of metastatic NSCLC (25 %), and 12 patients died of intercurrent disease (75 %). The causes of intercurrent death are summarized in Table 5. The estimated overall survival was 86 % at 1 year and 74 % at 2 years. The 2-year survival rate was 80 % for stage IA and was 65 % for stage IB (Fig. 3; *P* = 0.203). The cause-specific survival rate was 94 % at 1 year and 86 % at 2 years. The 2-year special disease survival rate was 93.3 % for stage IA and 75 % for stage IB (Fig. 4; *P* = 0.074).

**Table 5:** Characteristics of patients who died of intercurrent disease during follow-up

Sex	Age	Karnofsky performance status	Cause of death
Male	78	80	Cerebrovascular infarction
Male	82	70	Cerebrovascular infarction
Male	85	60	Myocardial infarction
Female	79	80	Myocardial infarction
Female	84	70	Myocardial infarction
Male	92	60	Respiratory failure
Female	89	70	Respiratory failure
Male	80	90	Traffic accident
Male	76	80	Sudden death
Female	83	70	Cardiac decompensation after dialysis
Male	65	70	Cardiac decompensation
Male	72	80	General deterioration

## Discussion

Radical radiotherapy is the suitable treatment for patients who refused to have operations or those who suffer from stage I NSCLC, but have contraindications for surgery because of objective reasons. Conventional radiotherapy improves the prognosis and increases the quality of life of patients. Although conventional radiotherapy improves the prognosis and quality of life, the dose is difficult to increase because the lung tumor target has low conformity during radiotherapy and it is influenced by respiratory motion. However, the development of CyberKnife provides a good solution to this problem. In this study, CyberKnife was used to treat 50 patients with stage I NSCLC, with an isodose for the tumor target prescription dose reaching 72 to 88 %. This treatment increased the target conformity and allowed the surrounding normal tissues to receive only a small dose of radiation. During the 3–45 months after treatment, few patients developed adverse reactions such as weakness, nausea, emesis, and bone marrow depression. These symptoms were relieved after proper treatments. The local control rate of the pulmonary lesions reached 92 % through regular follow-up and chest CT rechecking. Most of the patients achieved complete remission (the remission rate was 80 %). The quality of life was almost not influenced significantly.

The efficacy of conventional radiotherapy and three-dimensional conformal radiotherapy in stage I NSCLC is difficult to improve, which is mainly because real-time position tracking is impossible during treatment. CyberKnife SBRT with synchrony technology is one of the most mature real-time tracking technologies for clinical applications, and the relevant research results show accuracy up to around 1.5 mm [10]. Therefore, this technology reduces the uncertainty of tumor location through respiration adaptive tracking system and decreases the PTV caused by respiratory movement. This technology reduces the target volume, increases the effective dose, reduces the toxicity to normal tissues, and it significantly improves the efficacy [10–12]. Of the 50 patients in this study, all received synchronous respiratory tracking technique (synchrony) using gold seed fiducials implanted into the tumors. During the completion process of plans, 80 % of average isodose wrapped the target volume, with the CI reaching 1.17 and the nCI reaching 1.28. By increasing the tumor dose, CyberKnife significantly reduces the dose to normal tissues. The results show that CyberKnife needed only three times sessions in treating NSCLC, and the short-term efficacy was positive, with a total effective rate (CR + PR) of 92 %. The survival rate was 86 % at 1 year and 74 % at 2 years. The disease-specific survival rate was 94 % at 1 year and 86 % at 2 years. As a new exploration in the treatment of early-stage NSCLC, CyberKnife is similar to the surgery in terms of survival rate, and it has an increased local control rate.

Although the CyberKnife has its high accuracy, the border was still the problem during CyberKnife SBRT. A poorly controlled border range PTV could result in slight tumor metastasis, recurrence, or residues. During the planning of CyberKnife, defining the correct PTV range correctly is difficult. Timmerman [13] conducted the first test on increasing the dose of CyberKnife treatment for stage I NSCLC in 2003. The result showed that if the border was controlled within 1 cm, a dose of 60 Gy used three times in 2 weeks was safe. Moreover, Giraud [14] reported that 96 % of cases were had malignant tumor cells ranging from 6 to 8 mm beyond the GTV. Based on this result, Brown [15] extended the primary tumor from 8 to 10 mm and the metastatic tumor from 3 to 5 mm, achieving good results using CyberKnife SBRT. Thus, in the present study, we combined the residual inaccuracy of the synchrony and the micrometastatic characteristics of the tumor. PTV was defined as the border by extending the GTV to 8 mm to cover the border, which covers slight metastasis or unpredictable motion errors. From the results obtained from the 35 months of observation, the 2-year local control rate reached 96 %, which also showed that the boundary should be extended by 8 mm.

The problem of treatment dosage administration of CyberKnife SBRT has confused many experts. If the dose is too high, the tumor control rate is high, but the complications also increase. If the dose is too low, the complications are decreased, but the tumor control rate is low. Timmerman [16] treated 70 patients with stage T1 or T2 (≤7 cm) NSCLC with N0 and M0 pathology. The number of patients was 35 for both T1 and T2, including the peripheral and the central-type patients. The total dose of all patients was 60–66 Gy, divided into three sessions. The treatment was completed within 1–2 weeks. The results showed that the 2-year local control rate was 95 %, the median survival rate was 32.6 months, the 2-year overall survival rate was 54.7 %, and 28 patients died during follow-up. Among those treated for central lung cancer, five cases died of related cancer, six cases died of related treatment, and 17 cases died of related complications. For the patients with peripheral lung cancer, 83 % of patients did not have serious treatment-related toxicity within 2 years, but only 54 % of the central-type patients did not have serious toxicity. The results show that the peripheral lung cancers are well tolerated. Vander Voort vanzyp [17] treated 70 patients with stage I peripheral NSCLC in 2009, who were treated at 45 and 60 Gy, the division of which was completed in three times. Thus, the 2-year local control rate was 96 % at 60 Gy in 2 years, and the 45 Gy group was 78 % **(** *P* = 0.197). The 1-year overall survival rate was 83 %, whereas the 2-year overall survival rate was 62 %. The 60 Gy group did not have treatment-related complications more than grade III. The results from two authoritative studies showed that treating stage I peripheral NSCLC with 60 Gy in three divided doses is safe.

Based on these studies, the patients in our group were all stage I peripheral NSCLC. A dosage from 48 to 60 Gy was given for patients with poor lung function, but the total dose of 60 Gy was given for more than 60 % patients. The 2-year local rate was 96 % in three divided doses, similar to the reported studies [18]. However, the overall survival rate was 74 % in 2 years, slightly higher than that in the reported study [19]. Presumably, patient selection may play a role in the variation of overall survival.

The major symptoms of toxicity of CyberKnife SBRT include short-term weakness, nausea, and mild hematologic toxicity, which improved through active symptomatic treatment. The long-term adverse reactions are mainly local pain and local radiation pneumonia, which improved and returned to normal through positive symptomatic treatment. Long-term radiation therapy-related toxicity greater than grade III was not observed during the 2-year observation. The toxicity reaction increased with the prolongation of follow-up time. However, compared with other clinical studies [20] with median follow-up time of 12 months, we did not find any apparent increase in toxicity. However, further attention to clinical observations and follow-up is necessary to determine whether the long-term adverse reactions will occur.

The samples selected in the present study were stage I peripheral NSCLC, which has its limitations. CyberKnife SBRT is still a new technology, and clinical experience in the dosing and frequency of treatment for Asians is limited, especially in Chinese populations. During the treatment, we mainly referred to the clinical experience in North America and Europe, and the treatment modalities and doses were still in the exploratory stage. Central NSCLC limited the dose and the implantation of markers because of the proximity to the trachea and main bronchi. Thus, selecting treatment cases should be done more carefully.
